# High-quality draft genome and characterization of commercially potent probiotic *Lactobacillus* strains

**DOI:** 10.5808/GI.2019.17.4.e43

**Published:** 2019-11-07

**Authors:** Ayesha Sulthana, Suvarna G. Lakshmi, Ratna Sudha Madempudi

**Affiliations:** Center for Research and Development, Unique Biotech Limited, Hyderabad 500 078, India

**Keywords:** high-quality draft genome, *Lactobacillus*, probiotics, safety

## Abstract

*Lactobacillus acidophilus* UBLA-34, *L. paracasei* UBLPC-35, *L. plantarum* UBLP-40, and *L. reuteri* UBLRU-87 were isolated from different varieties of fermented foods. To determine the probiotic safety at the strain level, the whole genome of the respective strains was sequenced, assembled, and characterized. Both the core-genome and pan-genome phylogeny showed that *L. reuteri* was closest to *L. plantarum* than to *L. acidophilus*, which was closest to *L. paracasei*. The genomic analysis of all the strains confirmed the absence of genes encoding putative virulence factors, antibiotic resistance, and the plasmids.

## Introduction

*Lactobacillus* are a group of Gram-positive, rod-shaped, microaerophilic, non-spore-forming, lactic acid–producing bacteria [1], they are the natural and significant inhabitants of gastrointestinal tract of humans, as well as they are known to constitute a major part of the oral and vaginal microbiome [2-5]. *Lactobacillus* are the most common probiotics found in fermented food products, and the awareness of probiotic benefits is evolving more quickly. Commercially available *Lactobacillus* probiotic strains help to restore the microbiota of imbalanced gut caused due to antibiotic treatments; however, the pathogenicity and efficacy of potential probiotics have to be assessed for safety. Here, we report the whole genome sequence of commercially potent probiotic *Lactobacillus* strains: *Lactobacillus acidophilus* UBLA-34, *Lactobacillus paracasei* UBLPC-35, *Lactobacillus plantarum* UBLP-40, and *Lactobacillus reuteri* UBLRU-87.

*Lactobacillus* strains were isolated from serially diluted fermented foods under anaerobic conditions at 37℃ using MRS (deMan, Rogosa, and Sharpe) agar, the pure isolated colonies were cultured using MRS broth, the cells were harvested for DNA isolation with the phenol-chloroform extraction method, followed by 16S rRNA gene amplification (using the primers 27F and 1429R) [6], the strains were confirmed by PCR amplicons sequencing and phylogenetic analysis. High molecular weight genomic DNA of the identified strains was isolated by the above-described method, DNA fragments of 300- to 400-bp size were generated by ultrasonication, fragmented DNA was used to prepare a paired-end sequencing library with a Nextera DNA Flex Library preparation kit (Illumina, San Diego, CA, USA) and sequencing was performed on an Illumina NextSeq 500 System (Illumina).

A total of 2,735,462 (420× genome coverage), 2,213,461 (218× genome coverage), 2,337,040 (214× genome coverage), and 1,641,982 (270× genome coverage) paired-end reads were generated for *L. acidophilus* UBLA-34, *L. paracasei* UBLPC-35, *L. plantarum* UBLP-40, and *L. reuteri* UBLRU-87, respectively. The reads were quality filtered based on the Phred score using NGS QC Toolkit to remove low-quality sequences [7]. The quality-filtered paired-end reads were assembled to high-quality draft genomes (Table 1) by employing *de novo* genome assembler SPAdes version 3.11.1 [8] and the scaffolder SSPACE-standard version 3.0 [9].

The genomes were annotated using RAST [10] and the NCBI’s Prokaryotic Genomes Annotation Pipeline (PGAP) [11]. The genes were predicted and translated through the Prokaryotic Dynamic Programming Gene-finding Algorithm (Prodigal) program [12], following pathway identification with the Kyoto Encyclopedia of Genes and Genomes Automatic Annotation Server (KAAS) [13] (Table 2).

**Fig. 1.**
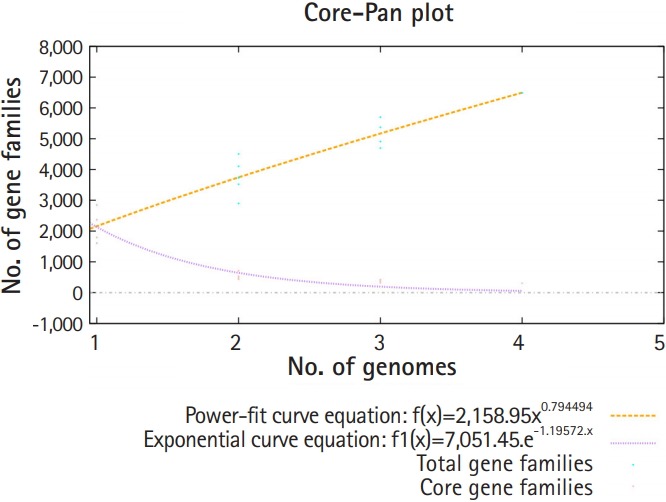
The pan and core genome plot of *Lactobacillus* strains (total gene families represented by black color, core gene families are denoted by pink color).

**Fig. 2.**
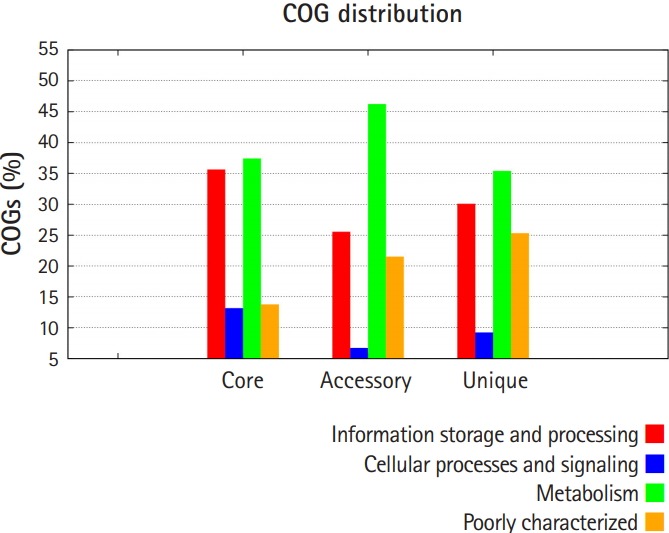
Cluster of orthologous groups (COG) distribution of the core, accessory and unique genes.

**Fig. 3.**
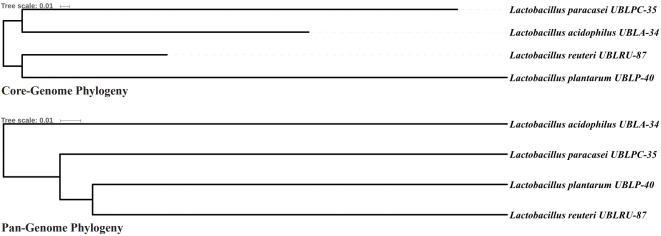
Core-Pan genome phylogeny.

Pan-genomic analysis of *Lactobacillus* strains was performed to determine the conserved core and variable genes (Table 3) [14], the estimated pan-genome size was 6,487, and the parameter ‘b’ was calculated to be 0.794494 (Fig. 1), which confirms that the pan-genome is open. The highest number of new genes which contributed to the pan-genome was observed for *L. plantarum* UBLP-40 (Table 3). The highest part of the core genome of *Lactobacillus* genus was composed of genes related to metabolism, the second-highest contributing genes were related to information storage and processing, whereas the unique and accessory genes contained more amount of poorly characterized genes in comparison to core genome (Fig. 2). The phylogeny of core and pan-genome showed that *L. reuteri* shares the relatedness with *L. plantarum*, whereas *L. paracasei* is closest to *L. acidophilus* (Fig. 3).

All the four genomes of *Lactobacillus* strains were screened to determine the presence of genes encoding for putative virulence factors such as hemolysin *BL*, non-hemolytic enterotoxin *NHE*, enterotoxin *T*, cytotoxin *T*, and cereulide [15], antibiotic resistance [16], and plasmids [17]. None of the genomes (UBLA-34, UBLPC-35, UBLP-40, and UBLRU-87) showed the presence of putative virulence factor or antibiotic resistance encoding genes or plasmids or any antibiotic-resistant genes containing plasmids. Secondary metabolite producing gene cluster detection was performed for all the *Lactobacillus* strains, based on the hidden Markov model profiling of metabolite producing genes [18].

## *Lactobacillus acidophilus* UBLA-34

**Fig. 4.**
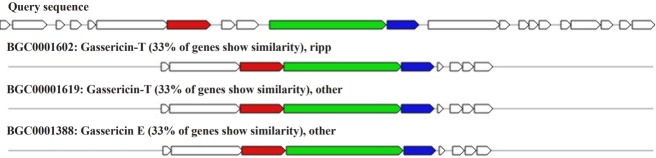
Bacteriocin gene clusters homologous to *Lactobacillus acidophilus* UBLA-34 (biosynthetic genes presented in red, regulatory genes in green and transport-related genes in blue color).

RiPP biosynthetic gene cluster was found in scaffold number 6 (location: 53,280–66,324 nt) consisting of seven genes encoding gassericin. The homologous gene cluster was mined from *Lactobacillus gasseri* LA327, gassericin T gene cluster *Lactobacillus gasseri* LA158 gassericin T gene cluster, *Lactobacillus gasseri* EV1461 gassericin E gene cluster with a 33% similarity (Fig. 4).

## *Lactobacillus paracasei* UBLPC-35

Two bacteriocin biosynthetic gene clusters were found in scaffold number 1 (location: 21,360–44,300 nt and 85,659–97,824 nt), there was no significant similarity found with the known gene clusters.

## *Lactobacillus plantarum* UBLP-40

First bacteriocin biosynthetic gene cluster was found in scaffold number 7 (location: 101,210–113,360 nt), whereas terpene biosynthetic gene cluster was found in scaffold number 12 (location: 77,136–92,747 nt), there was no significant similarity found with the known gene clusters.

## *Lactobacillus reuteri* UBLRU-87

No secondary metabolite producing gene cluster was found.

## Data Availability

The raw sequence reads have been submitted to the NCBI SRA and the whole-genome shotgun project has been deposited in DDBJ/EMBL/GenBank under the following accession numbers: *Lactobacillus acidophilus* UBLA-34: SRR7958229, RBHY00000000: the version described in this paper is version RBHY01000000, *Lactobacillus paracasei* UBLPC-35: SRR8382560, RCFI00000000: the version described in this paper is version RCFI01000000, *Lactobacillus plantarum* UBLP-40: SRR8382543, RDEY00000000, the version described in this paper is version RDEY01000000, *Lactobacillus reuteri* UBLRU-87: SRR8382542, RIAU00000000, the version described in this paper is version RIAU01000000.

## Figures and Tables

**Table 1.** Genome characteristics

Strain	Genome size (bp)	No. of scaffolds	Largest scaffold size (bp)	N50 (bp)	GC (%)
UBLA-34	1,951,037	34	669,777	167,656	34.6
UBLPC-35	3,038,799	11	2,520,091	2,520,091	46.02
UBLP-40	3,265,595	47	528,446	245,973	44.49
UBLRU-87	1,821,307	21	1,763,886	1,763,886	38.55

**Table 2.** Genome annotation

Subsystem feature counts	UBLA-34	UBLPC-35	UBLP-40	UBLRU-87
Cofactors, vitamins, prosthetic groups, pigments	45	56	106	82
Cell wall and capsule	28	47	60	38
Potassium metabolism	5	3	7	5
Membrane transport	42	49	53	19
Iron acquisition and metabolism	4	7	5	5
RNA metabolism	31	35	39	35
Nucleosides and nucleotides	78	83	88	82
Protein metabolism	122	132	136	130
Cell division and cell cycle	4	5	4	5
Regulation and cell signaling	23	34	29	10
Secondary metabolism	1	4	4	1
DNA metabolism	47	74	56	49
Fatty acids, lipids, and isoprenoids	23	47	35	46
Nitrogen metabolism	0	4	9	9
Dormancy and sporulation	5	6	6	5
Respiration	12	28	16	15
Stress response	5	46	20	8
Amino acids and derivatives	91	122	196	110
Sulfur metabolism	4	5	3	3
Phosphorus metabolism	15	28	33	28
Carbohydrates	124	233	240	115
Coding sequences	1,897	3,156	3,214	1,832
No. of RNAs	63	59	70	72

**Table 3.** Pan-genome analysis

Strain	No. of accessory genes	No. of unique genes	No. of exclusively absent genes	No. of core genes
UBLA-34	364	1,119	118	308
UBLPC-35	484	1,577	105	308
UBLP-40	746	1,792	12	308
UBLRU-87	513	787	64	308
